# Editorial: Financial Intermediation Versus Disintermediation: Opportunities and Challenges in the FinTech Era

**DOI:** 10.3389/frai.2020.629105

**Published:** 2021-01-14

**Authors:** Meryem Duygun, Shatha Qamhieh Hashem, Alessandra Tanda

**Affiliations:** ^1^ Nottingham University Business School, University of Nottingham, Nottingham, United Kingdom; ^2^ Department of Banking and Financial Sciences, An-Najah National University, Nablus, Palestine; ^3^ Department of Economics and Management, University of Pavia, Pavia, Italy

**Keywords:** FinTech, disintermediation, finance and credit sector, peer to peer, ICOS, cryptocurrencies, robo advisors, risk management

Financial Technology (FinTech) emerged in the 21st century as a significant and innovative force that profoundly disrupts the traditional financial intermediation channels. FinTech has changed both the way financial products are produced and how they are distributed. Technology extensively used by FinTech has been a driver for improving and automating the business model for banks and other financial institutions. The combination of modern technological advancements and financial products and services challenged the traditional financial industry to provide better financial solutions to their clients. Equally FinTech set further pressure by allowing non-financial businesses to provide tailored digitalized financial solutions. Thus, the traditional workflow of the financial industry is currently being disrupted by FinTech and by its incumbents.

This special issue “Financial intermediation vs. disintermediation: Opportunities and challenges in the FinTech era” targets the improvements and challenges that emerged with FinTech. It collects valuable contributions for the interested audience in understanding and analysing the FinTech phenomenon from multiple perspectives.

In this issue, the study contributed by Omarini (“FinTech: a new hedge for a financial re-intermediation. Strategy and risks perspectives”) emphasizes that new entrants and technologies are changing dramatically the banking process from an isolated silo approach to an open banking approach. Here the traditional banking business model is questioned as new ways of innovation, cooperation are arising in a manner that is altering the conceptualization of conventional banking business models. New entrants are putting pressure on the traditional banking processes by offering innovative cost efficient financial services and products through the use of lean structures and an appealing seamless customer experience.

Another contribution by Brandl and Hornuf titled “Where Did FinTechs Come From, and Where Do They Go? The Transformation of the Financial Industry in Germany After Digitalization” investigates how the financial industry is being transformed by FinTech companies in Germany. The study points out that traditional participants in the financial system are trying to approach the digitalization of their business with various strategies and different degrees of success. The authors indicate that there is an increase in the number of strategic partnerships established between traditional financial agents and FinTech. Nevertheless banks and financial institutions remain reluctant in opening up their structures to FinTech, often because of the difficulties that they face due to their different IT standards and infrastructure. Thus they are partially participating in the digitalization surge. The authors pointed out the major attention dedicated by academics and observers to the emerging trend of cryptoassets, i.e. Bitcoin. Cryptocurrencies and cryptoassets were proposed initially as an alternative to the legal tender (currencies) which created some worries at the supervisory level. Worth to note that they showed an important development, but remained not so widely spread. This is because of the possibilities introduced by these assets on the market. Overtime, this contributed to a decrease in the threat to the normal currencies, but an increase of cryptoassets in the world of investment.

An additional contribution to the special issue is provided by Baldan and Zen “The Bitcoin as a Virtual Commodity: Empirical Evidence and Implications”. The focus of this study is on the role of distributed ledger technology (DLT) and of Bitcoin, and particularly on its pricing dynamics. The virtual coin shows an unpredictable pattern in most of the cases with high volatility. The evidence provided by the authors signals the need of further research on this issue. They also suggest to explain the Bitcoin prices not only with the profit and cost function, but also with additional explanatory parameters including technical features, i.e. the internet, and financial variables, i.e. financial indexes.

Using data drawn from the same asset class, a contribution is presented by Giudici et al. (“Network Models to Enhance Automated Cryptocurrency Portfolio Management”). This study provides an original approach to build efficient portfolio allocation strategies including cryptoassets and other volatile financial instruments. By means of a network model, they reach enhanced portfolio results in terms of performance and risk compared to what was obtained through more traditional models.

Cryptocurrencies can also be used to raise money similarly to what happens during an IPO. The Initial Coin Offerings (ICO) are a way for companies to raise funds through a cryptocurrency linked to the company. The surge of ICO has been accompanied by fraudulent initiatives and this has determined important losses. Toma and Cerchiello investigate this matter in their contribution titled “Initial Coin Offerings: Risk or Opportunity?”. By analysing the Telegram chats, white papers and websites through sentiment analysis, the authors show that several elements convey a positive sentiment that helps in detecting genuine ICOs from those that are not so.

Another area that has been transformed by FinTech is the area of lending. The technological development brought new entrants to offer peer-to-peer lending services or lending by crowdfunding platforms. Nevertheless, the financial and banking sectors can employ such technological developments as financing and lending alternatives, not only in terms of capitalizing on the new forms of intermediation in the credit industry, but also in terms of benefiting from such improvements in the process of lending. Furthermore, the FinTech new technologies can boost the predictive power of traditional models in the estimation of credit risk. The paper presented by Cerchiello and Scaramozzino (2020) “On the Improvement of Default Forecast Through Textual Analysis” provides an interesting example. Through the use of text mining on a sample of transactions carried out by borrowers, the authors propose an augmented model for credit risk scoring and show that the approach produces interesting results in terms of improved accuracy with respect to the baseline model.

More sophisticated estimation techniques and big data models might not necessarily call for process and results “unexplainability” as maintained by Bussmann et al. Their paper “Explainable AI in FinTech Risk Management” provides an application of sophisticated estimation techniques that employ artificial intelligence, but still have the characteristic of being explainable. This is particularly appreciable in the financial industry, where many stakeholders need to understand not only the result of a process (e.g., credit scoring, investment recommendation, etc.) but also the process itself and its implications. These stakeholders include investors, consumers, regulators and supervisory authorities.

Asset management is another segment of financial services that has benefited from the technological changes over time such as the developments in electronic trading. In the new wave of FinTech transformation, asset management is being electronically integrated within a number of important applications. Among these is the tool of automated advisory services which is becoming more widespread, despite that it has received - so far - less attention than other services, as in the case of lending or payments.

The paper provided by Boreiko and Massarotti on “How Risk Profiles of Investors Affect Robo-Advised Portfolios” investigates the investment suggestions provided to investors by 53 different digital advisors. They find that robo-advisory services, although formally complying with regulatory provisions on investments, show high variability in the investment recommendations even for the same risk-type model investor. Their results underline the need for a harmonised approach by regulators on this innovation that is introduced by FinTech.

The FinTech phenomenon has many facets and every facet presents its novelty, its advantages and risks that investors, customers, industries, policy makers and regulators have to consider. This special issue provides insights on some aspects of FinTech innovation, ranging from the most successful ones (such as peer-to-peer lending and cryptoassets) to more specific technological innovations and modelling approaches that move forward the knowledge and understanding on the FinTech phenomenon. We remark that while this special issue represents a significant contribution to the existing FinTech literature, still more research on the themes of the special issue will be needed to develop new methodologies and tools to enhance the understanding of the FinTech phenomenon.

## Author Contributions

MD, SH, and AT contributed to the design of the editorial and to the writing of the manuscript.

## Conflict of Interest

The authors declare that the research was conducted in the absence of any commercial or financial relationships that could be construed as a potential conflict of interest.

The handling editor declared a shared affiliation with one of the authors AT at time of review.

